# Association of Sleep, Inflammation and Female Infertility: A Cross‐Sectional Survey and Genetic Approach

**DOI:** 10.1002/brb3.70627

**Published:** 2025-06-17

**Authors:** Xin Xin, Jiaxi Li, Jinfu Zhang, Haicui Wu

**Affiliations:** ^1^ Guanghua Hospital Affiliated to Shanghai University of Traditional Chinese Medicine Shanghai China; ^2^ Shanghai University of Traditional Chinese Medicine Shanghai China; ^3^ Shandong Wendeng Osteopathic Hospital Wendeng China; ^4^ Affiliated Hospital of Shandong University of Traditional Chinese Medicine Jinan China

**Keywords:** genetic association, infertility, inflammation, Mendelian randomization, NHANES

## Abstract

**Introduction:**

Inflammation has been implicated in both reproduction and sleep; however, the relationships remain unclear, and conclusive evidence for genetic associations is lacking.

**Methods:**

This study utilized the 2013–2020 National Health and Nutrition Examination Survey (NHANES) data and conducted a Mendelian randomization (MR) analysis to investigate the associations among inflammation, sleep, and female infertility. A cross‐sectional study was performed on 370 infertile women aged 20–44 years. Multiple linear regression was applied to evaluate the associations between sleep and the inflammatory markers. Subgroup and interaction analyses were conducted to assess the robustness of the findings. MR analysis was performed on 91 circulating inflammatory proteins, 11 sleep traits, and infertility to examine potential genetic associations.

**Results:**

A total of 370 infertile participants aged 20–44 years were included. The logarithmic platelet–lymphocyte ratio (PLR) was significantly higher in the sleep disorder group (*p* < 0.05). After adjusting for potential confounders, sleep disorders remained associated with a reduction in PLR (*β* = −0.145, 95% CI: −0.267 to −0.023, *p* < 0.05). MR analysis using the inverse variance weighted (IVW) method indicated that insomnia, subjective long sleep duration, and high sleep efficiency were associated with increased levels of oncostatin‐M, artemisinin, and adenosine deaminase, all of which are implicated in infertility. Additionally, the morning type was associated with increased levels of C‐X‐C motif chemokine 5, which reduced the risk of infertility.

**Conclusion:**

Sleep is associated with various inflammatory factors in the body and may contribute to infertility. Inflammation appears to play a key role in mediating the complex interplay between sleep and reproduction. These findings highlight the potential value of screening and managing specific inflammatory markers in infertile patients with sleep disorders to improve reproductive outcomes. However, further clinical studies and mechanistic experiments are needed to validate the genetic associations identified in this study.

## Graphical abstract

**The association between sleep, inflammation, and female infertility is explored in this study**. Preliminary results from a cross‐sectional study involving 307 female infertility participants, drawn from the NHANES database (2013–2020), indicated that women with sleep disorders exhibit abnormal inflammatory states, particularly manifested as altered platelet‐to‐lymphocyte ratios. Mendelian randomization analysis, leveraging genetic data from 11 sleep traits, 91 circulating inflammatory proteins, and infertility within the GWAS database, further highlighted the potential mechanisms through which insomnia, sleep duration, sleep efficiency, and chronotypes may influence infertility by modulating specific inflammatory protein levels.

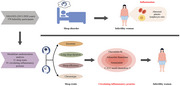

**Abbreviations**
ADA: adenosine deaminase
BMI: body mass index
CI: confidence interval
CXCL5: chemokine C‐X‐C motif ligand 5
GWAS: genome‐wide association study
IL‐6: interleukin‐6
IV: instrumental variable
IVW: inverse variance weighted
L5 timing: at least 5 h of active timing
LD: linkage disequilibrium
LIFR: leukemia inhibitory factor receptor
LMR: lymphocyte–monocyte ratio
MR: Mendelian randomization
MR‐PRESSO: Mendelian randomization‐pleiotropy residual sum and outlier
NHANES: National Health and Nutrition Examination Survey
NLR: neutrophil–lymphocyte ratio
nSNP: number of SNPs
OSM: oncostatin‐M
PID: pelvic inflammatory disease
PIR: poverty‐to‐income ratio
PLR: platelet–lymphocyte ratio
SII: systemic immune inflammatory index
SNP: single nucleotide polymorphism
SPT: sleep period time
WM: weighted median

## 1 Introduction

The global fertility rate has declined by more than 50%, particularly in high‐income countries, with 75% of these nations projected to have a below‐replacement fertility rate by 2050 (GBD 2021 Fertility and Forecasting Collaborators 2024). Factors contributing to this decline include the improvement in women's educational attainment, changes in fertility concepts, and infertility caused by various risk factors, all of which have delayed women's active childbearing decisions (L. Li et al. 2024; S. F. Wang and Seifer, 2024). Studies have shown that 17% of women and 15% of men report fertility issues, encompassing a wide range of diseases that affect both individual and couple fertility (Skåra et al. 2022; Zegers‐Hochschild et al. 2017).

Chronic inflammation in the female reproductive system, including systemic immune responses mediated by immune cells and chemokines, has long been associated with poor follicle quality and fertility challenges (Park et al. 2024; Fereidouni et al. 2024; Omidvar‐Mehrabadi et al. 2024; Gica et al. 2020). Reducing inflammation in follicular fluid, granulosa cells, and the endometrium has emerged as an important strategy to improve fertility outcomes (Shirvanizadeh et al. 2024; Wei et al. 2024). Emerging inflammatory markers, such as the systemic immune inflammatory index (SII), platelet–lymphocyte ratio (PLR), neutrophil–lymphocyte ratio (NLR), and lymphocyte–monocyte ratio (LMR), are calculated based on peripheral blood cell counts. Cross‐sectional studies have demonstrated associations between these markers and female infertility (Y. Chen, Xu, et al. 2024). Additionally, elevated levels of circulating inflammatory proteins, including macrophage colony‐stimulating factor and growth‐regulated oncogene‐alpha, have been linked to increased risks of female infertility and reproductive disorders (Lin et al. 2023).

The mutual mechanisms linking sleep traits and immune regulation hold therapeutic potential for inflammation and other diseases (Irwin, 2019; Irwin et al. 2023). Insomnia has been identified as a predictor and potential regulator of systemic inflammation (Ballesio et al. 2024). Sleep apnea syndrome has also been associated with elevated levels of circulating inflammatory proteins (Y. Zhao et al. 2025). Studies have shown that sleep disorders increase markers such as C‐reactive protein and the NLR level (Ma et al. 2024; Yin et al. 2023).

Sleep is also closely related to fertility (Z. Wang, Lai, et al. 2024). Poor sleep quality has been associated with reduced ovarian reserve in women of childbearing age (Lin et al. 2024). Shorter sleep duration, delayed sleep onset, and snoring are additional risk factors for premature ovarian failure (Cai et al. 2024). A prospective cohort study found that longer sleep duration is positively correlated with the success rate of embryo transfers (Caetano et al. 2021). Sleep problems and unhealthy sleep patterns in infertile women significantly reduce the quantity and quality of oocytes, as well as fertilization and clinical pregnancy rates (Q. L. Li et al. 2023). The circadian rhythm system is also implicated in miscarriage and recurrent miscarriage, likely due to abnormal expression of circadian rhythm genes that disrupt endometrial decidualization and embryo implantation (Roberto et al. 2024; Luo et al. 2024; Cui et al. 2022). Melatonin's role in improving reproductive outcomes by reversing inflammation and promoting decidualization underscores the importance of sleep disorders and inflammation in female fertility (Cui et al. 2021). Animal studies have further demonstrated that sleep deprivation causes cellular oxidative stress and mitochondrial dysfunction, leading to infertility by altering the transcriptome and metabolome of oocytes (Yi et al. 2024).

Inflammatory responses are believed to mediate the interaction between sleep and reproductive system dysfunction (Zheng et al. 2024). While existing studies have explored these associations, the causal relationships remain unclear due to limitations such as reverse causality and confounding factors, which can bias research findings. Genetic variation data for 91 circulating inflammatory proteins provide a valuable tool for comprehensively linking sleep and female fertility (Lee and Lim, 2019). Mendelian randomization (MR) analysis, which leverages genetic exposure, optimizes subsequent randomized trial designs (Ference et al. 2021) and serves as a reliable method for exploring causal relationships after cross‐sectional studies (Dudbridge, 2021; He et al. 20241). The random distribution and variation of genetic traits from conception prevent interference from confounding factors, disease progression, or reverse causality.

This study aims to use data from the National Health and Nutrition Examination Survey (NHANES) to investigate the associations among sleep disorders, inflammatory factors, and infertility. MR analysis will be conducted to explore whether different sleep characteristics are directly or indirectly related to infertility through inflammatory pathways.

## 2 Materials and Methods

### 2.1 Research Design

**FIGURE 1:**
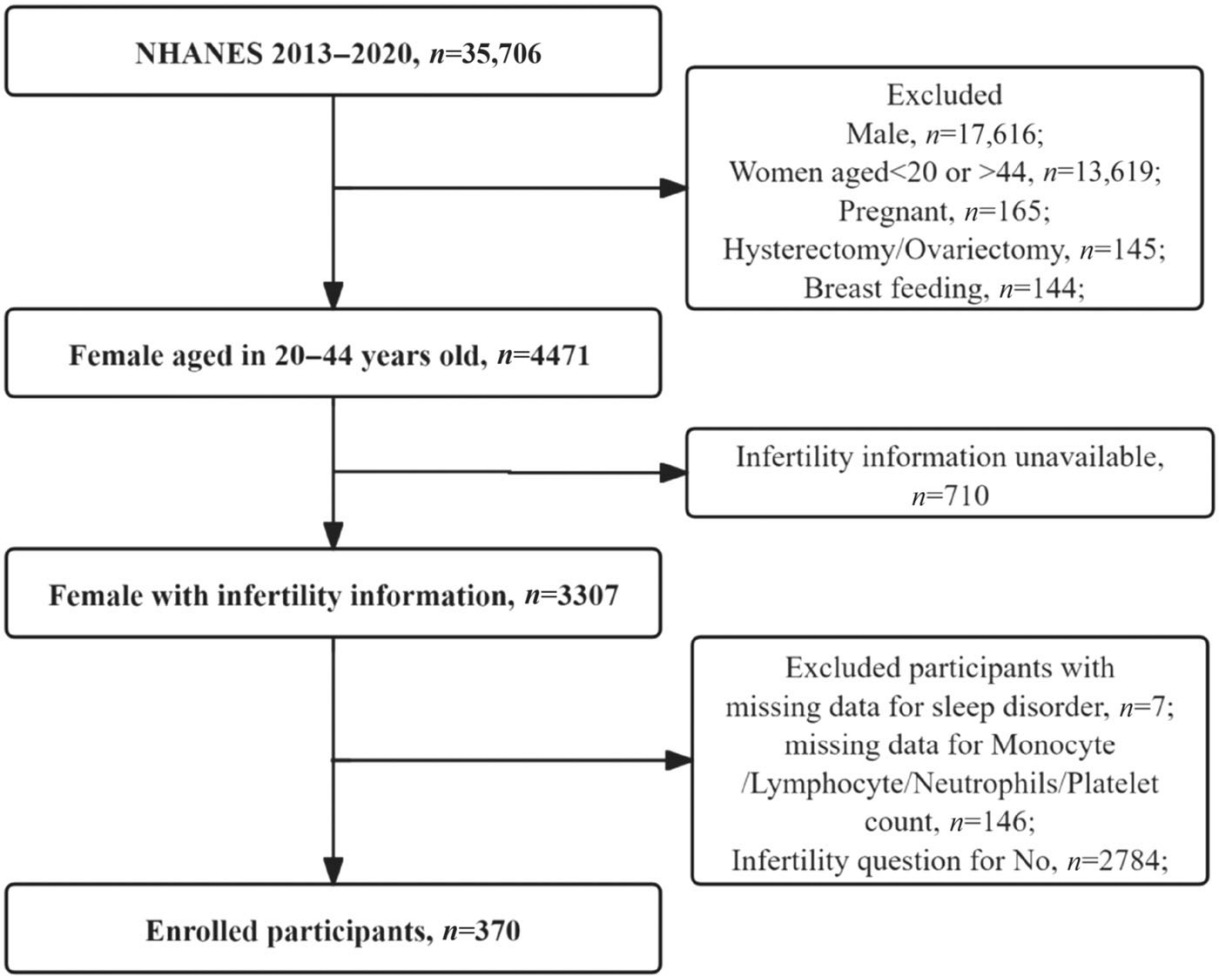
Flowchart of the inclusion and exclusion criteria for selecting participants from the NHANES. NHANES: National Health and Nutrition Examination Survey.

Figure 1 provides an overview of the research design. The first part of the study investigated the relationship between sleep disorders and circulating inflammatory markers, including SII, PLR, NLR, and LMR levels, in infertile women, which was achieved through linear regression analysis, subgroup analysis, and interaction analysis using data from the NHANES database of 2013–2020. In the second part, we explored the genetic associations of 11 sleep features, including accelerometer‐based sleep duration, chronotype, daytime napping, daytime sleepiness, insomnia, L5 timing (at least 5 h of active timing), the number of sleep episodes, self‐reported short sleep, self‐reported long sleep, self‐reported sleep duration, and sleep efficiency, 91 circulating inflammatory proteins, and female infertility based on the genome‐wide association study (GWAS) database through multiple bidirectional MR analyses to further evaluate the causal relationship of genetic instrumental variables (IVs) on inflammatory factors in infertile women with sleep disorders.

### 2.2 Data Sources

The NHANES is conducted by the National Center for Health Statistics of the United States and is based on questionnaires, physical examinations, family interviews, and laboratory tests. On the basis of a large cross‐sectional survey of the US population, multistage stratified probability sampling ensured that the samples were highly representative. The data used in this study were sourced from 2013 to 2020, and all participants provided written informed consent in accordance with the NHANES protocol. Approval from the relevant ethics review committee was obtained. The data used in this study are from https://www.cdc.gov/nchs/nhanes. In addition, we used two‐sample MR analysis to evaluate the causal relationships among 91 inflammatory factors, sleep characteristics, and female infertility. In addition, datasets including other diseases or treatments unrelated to the study were excluded. All GWASs were approved by the relevant ethics review committee, and all participants provided written informed consent. Supporting Information S1 provides detailed information on the GWAS dataset.

#### 2.2.1 Data Sources for GWASs of Sleep Traits

We examined 11 sleep‐related traits, and each sleep trait was obtained from a published large‐scale GWAS of people of European descent from the UK Biobank. Self‐reported sleep duration refers to the total sleep duration (including naps) over a 24‐h period, and all participants reported their sleep duration as integer values adjusted for age and sex. In addition, participants reported 7–8 h of overall sleep time as a control, with short overall sleep time defined as ≤ 6 h and long sleep time defined as ≥ 9 h. Notably, all participants who self‐reported using a sleep aid and had an overall sleep duration of less than 3 h or more than 18 h were excluded (Dashti et al. 2019).

Chronotype refers to the tendency to get up early, get up late, or somewhere in between. In this study, the case group included participants who self‐reported their chronotype as “definitely morning” or “more morning,” whereas those who self‐reported as “definitely evening” or “more evening” were placed in the control group, with data adjusted for age, sex, and other factors (Jones, Lane, et al. 2019).

Data on daytime napping were obtained by asking participants if they nap during the day (Dashti et al. 2021), and data on daytime sleepiness were obtained by asking the participants, “How likely is it that you don't want to take medication or fall asleep during the day (for example, while working, reading, or driving)?” adjusting primarily for self‐reported age and sex, as well as factors such as body mass index (BMI) (H. Wang et al. 2019). Insomnia is defined as difficulty falling asleep at night or waking up in the middle of the night after falling asleep (Lane et al. 2019).

The characteristics of the accelerometer‐based sleep measurements included total sleep duration, L5 time, sleep frequency, and sleep efficiency, which are more objectively estimated on the basis of the sleep period time (SPT) window, which is determined via actigraphy devices. Sleep duration was defined as the total sleep duration within the SPT window, and participants with a total sleep duration of less than 3 h or more than 12 h were excluded. The L5 time was defined as the midpoint of the 5‐h period in which the average acceleration was least active each day. The number of nightly sleep episodes was defined as the number of sleep episodes that lasted at least 5 min within the SPT window. Participants with an average frequency ≤ 5 or ≥ 30 were excluded. Sleep efficiency was defined as the sleep duration divided by the SPT window duration (Jones, van Hees, et al. 2019).

#### 2.2.2 GWAS Data Sources for Inflammatory Factors

Genetic variation data for 91 inflammatory proteins were obtained from GWASs. Genome‐wide protein quantitative trait locus mapping was performed for 91 circulating inflammatory factors via the Olink Target‐96 platform (Thermo Fisher Scientific, USA). A meta‐analysis of the aggregated data for each cohort was performed (J. H. Zhao, Stacey, et al. 2023).

#### 2.2.3 GWAS Data Sources for Infertility

We used infertility‐related genetic variation data from the FinnGen database released in 2021 with female sterility phenotype summary data.

### 2.3 Study Population

We screened a total of 35,706 participants from 2013 to March 2020. Following previous research (J. Zhao, Fu, et al. 2023), we initiated the study by excluding participants who were male (*n* = 17616), under 22 years of age or over 40 (*n* = 13619), pregnant (*n* = 165) or breastfeeding (*n* = 144) at the time of the survey, or had undergone hysterectomy or oophorectomy (*n* = 145). Next, participants with missing data on infertility status (*n* = 710), sleep disorders (*n* = 7), or inflammatory markers (*n* = 146) were also excluded. Finally, we included only those participants who met the inclusion criterion of “attempting to conceive for at least one year without achieving pregnancy,” as indicated in the survey questionnaire. This process resulted in a final sample of 370 infertile female participants aged 20–44. The detailed criteria and exclusion process are illustrated in Figure 1. Additional data collected on the 370 participants included information on menstrual cycle, history of pelvic infections, lifestyle habits, and chronic diseases such as diabetes and hypertension. These variables were incorporated into the follow‐up analysis to assess whether they exerted any confounding effects.

### 2.4 Exposure and Outcome Variables

Sleep disorders were considered as exposure variables and were defined when “YES” was answered in response to the DPQ30 question—“trouble sleeping or sleeping too much” in the Mental Health Depression Screener section of the survey questionnaire. The levels of the inflammatory markers SII, PLR, NLR, and LMR were used as outcome variables. A total of 1000 cells/µL of lymphocytes, platelets, neutrophils, and monocytes were collected from a complete blood to calculate the following inflammatory markers: SII = platelet × neutrophil/lymphocyte, PLR = platelets/lymphocytes, NLR = neutrophils/lymphocytes, and LMR = lymphocytes/monocytes.

### 2.5 Covariant Quantity

In accordance with previous studies (L. Wang, Bai, et al. 2024; Y. Zhao et al. 2024) and clinical practice experience, we selected age, race, marital status, education level, BMI, the family poverty‐to‐income ratio (PIR), menstrual status, history of pelvic inflammatory disease (PID), smoking, drinking, diabetes, hypertension, sedentary behavior, and physical activity as the covariates. According to clinical significance, age was divided into 20–30, 31–40, and > 40 years; race was divided into Mexican American, non‐Hispanic White, non‐Hispanic Black, non‐Hispanic Asian, and others; marriage status was divided into married/living with a partner and divorced/separated/never married/widowed; education level was divided into below high school, high school or equivalent, and college or above; and BMI was divided into < 25, 25–30, and ≥ 30 (Xia et al. 2023). The PIR was recommended by the NHANES official website to be divided into low incoming level (≤ 1.3), middle incoming level (1.3–3.5), and high incoming level (≥ 3.5). Sedentary behavior was classified on the basis of whether the sitting time was ≥ 360 min (Zhang and Liu, 2024); physical activity was classified on the basis of whether the total duration of moderate‐ and high‐intensity exercise on a typical day exceeded 30 min (Bloomberg et al. 2024); PID and menstrual cycle regularity were defined on the basis of the answers to the reproductive health questionnaires RHQ031 and RHQ078. Smoking status was classified on the basis of “whether you have smoked at least 100 cigarettes in your lifetime” and “whether you smoke now”: “Never” was defined as not exceeding 100 cigarettes in your lifetime, “Before” was defined as exceeding 100 cigarettes in your lifetime but not smoking now, “Now” was defined as smoking almost every day or some days. Drinking was classified on the basis of “having consumed at least 12 glasses of alcohol in lifetime or having consumed any type of alcohol in lifetime” and “how often drinking alcohol over past 12 months”: “Never” was defined as having consumed no more than 12 glasses in lifetime or having never consumed alcohol in lifetime, “Before” was defined as having consumed more than 12 glasses in lifetime or having consumed alcohol but not consumed more than 12 glasses within 12 months, “Now” was defined as drinking more than 12 drinks within 12 months. Diabetes was determined according to “doctor told you have diabetes,” insulin use, and diabetes drug use. Hypertension was determined on the basis of “doctor told you have high blood pressure 2+ times” and “now taking prescribed medicine for HBP.”

### 2.6 Selection of Genetic IVs

**FIGURE 2:**
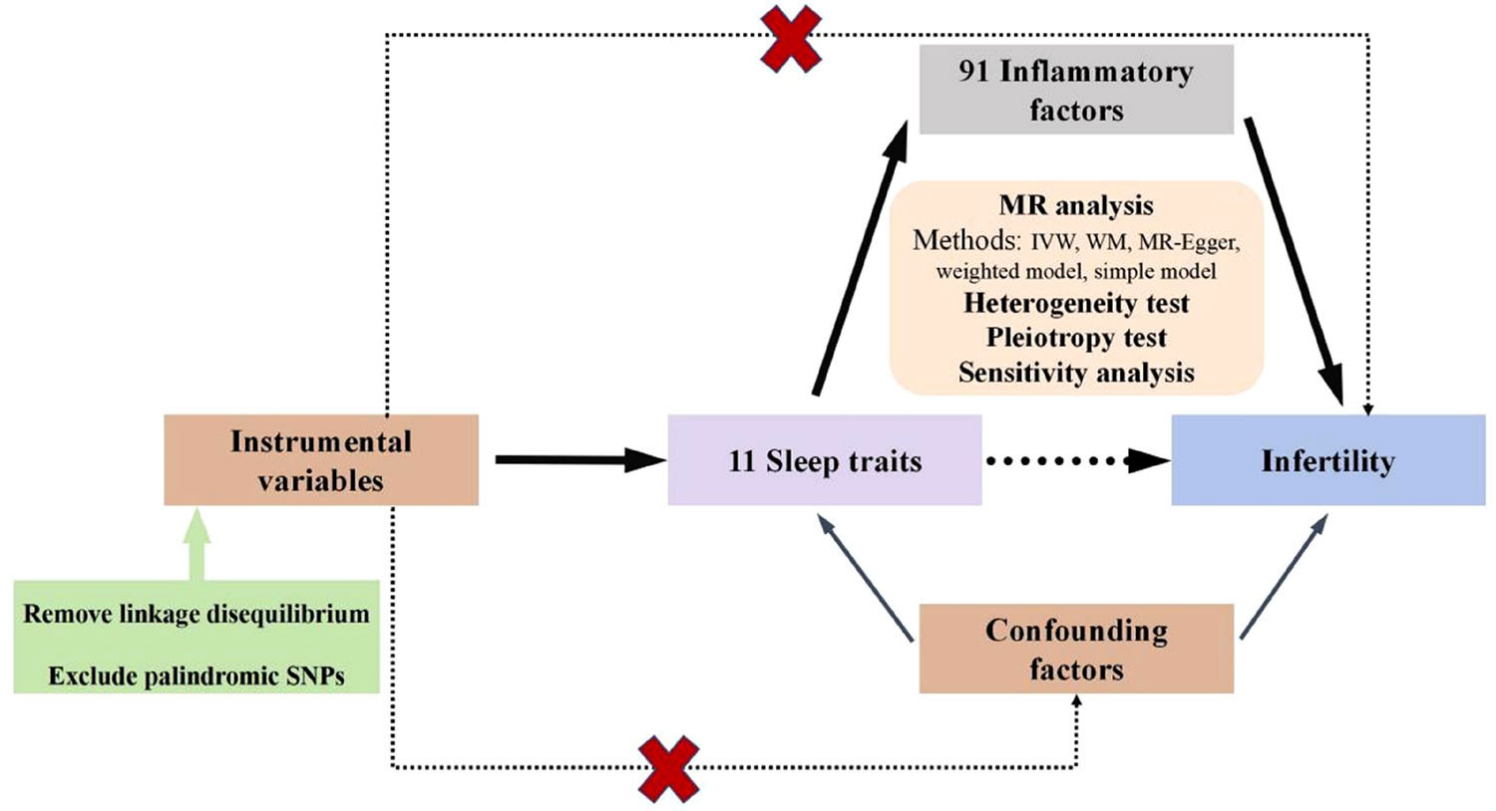
Hypotheses and study design of MR studies on the associations among sleep traits, inflammatory factors, and infertility. MR: Mendelian randomization; SNP: single‐nucleotide polymorphism; IVW: inverse‐variance weighted; WM: weighted median.

A visual representation of the two‐sample MR analysis is shown in Figure 2. The genetic IVs of MR studies must first satisfy the following three hypotheses: (1) they are closely related to exposure; (2) they are independent of the outcome and any known or unknown confounders; and (3) they affect results only through exposure (Burgess et al. 2017).

We determined the optimal gene IV for inflammatory factors and sleep‐related characteristics on the basis of strict criteria (Hu et al. 2023). First, *p* < 5e‐8 was used as the screening criterion to identify single nucleotide polymorphisms (SNPs) with significant genome‐wide associations. Second, SNPs with linkage disequilibrium (LD) *r* ^2^ > 0.001 in the 10,000 kb window were removed to ensure the independence of the IVs. Third, the *F*‐statistic (*F* = *β* ^2^/SE^2^) was used to calculate the statistical strength of the IVs. SNPs with *F* < 10 were considered to have weak statistical strength, and they were excluded to avoid bias. If there were no SNPs in the outcome of the exposure, we looked for proxy SNPs with an LD *r* ^2^ > 0.80 and ignored the exposure if there were no proxy SNPs. Palindromic and incompatible SNPs that could not be determined were eliminated. PhenoScanner (http://www.phenoscanner.medschl.cam.ac.uk/) was then used to rule out confounding factors or outcome‐related SNPs (Kamat et al. 2019). In addition, considering that some of the biological information regarding SNPs was not clear, on the basis of the screening results obtained via PhenoScanner, we included statistical analysis methods to detect pleiotropy to eliminate the pleiotropy of genetic IVs.

### 2.7 MR Analysis

The MR analysis used inverse variance weighted (IVW) analysis, weighted median (WM) analysis, MR‒Egger regression, a weighted model, and a simple model to evaluate the causal relationship. IVW analysis was the main statistical analysis method used (Burgess et al. 2013; Bowden et al. 2016), and *p* < 0.05 was considered statistically significant. The intercept test of the MR‒Egger method was adopted to analyze whether the IVs had a pleiotropic effect on the results and provided estimates of causal effects independent of direct effects under weak assumptions (Burgess and Thompson, 2017). Weighted‐ and simple‐model methods have also been used for sensitivity (M. Xiang, Wang, et al. 2023). The weighted model clusters the SNPs on the basis of similarity and statistics of the inverse variance weighting of the SNPs of each cluster. A causal estimate was derived on the basis of the maximum weighting (Hwang et al. 2019).

Scatter, forest, and funnel plots were used to visualize the MR results. Additionally, we used the Mendelian randomization‐pleiotropy residual sum and outlier (MR‐PRESSO) method to test all the SNPs globally (Verbanck et al. 2018). When SNPs with heterogeneity were identified, the IVW random effects model was applied (Yang et al. 2022), and a *p* value < 0.05 was considered statistically significant. The MR‒Egger intercept was used to evaluate horizontal pleiotropy (Lu et al. 2024). Finally, “leave‐one‐out” analysis was conducted to test the sensitivity of the SNP's influence on the outcome. The combined effect of the remaining SNPs was calculated by removing a single SNP to confirm that no causality was driven by a single SNP, thus reducing bias (X. Chen, Hao, et al. 2024). We did not correct for multiple testing in this exploratory study to discover more potential positive indicators (Y. Xiang, Zhang, et al. 2023; Zhang et al. 2022).

### 2.8 Statistical Analysis

Interpolation of missing data for all covariates was performed to reduce bias. We selected the weights that reflect the least number of people and used the Mobile Test Center Exam Weight (WTMEC2YR) to calculate the sampling weights for 2013–2016 and 2017–March 2020 and applied them to each subsequent analysis. The categorical variable is represented as the actual frequency (weighted proportion %), and the continuous variable is represented as the WM (interquartile range). The chi‐square test or Wilcoxon test was used to compare the differences in categorical and continuous baseline variables between two groups. To avoid the influence of extreme values, log_2_ conversion was performed on the SII, PLR, NLR, and LMR.

We used linear regression with two different models to investigate the independent associations between sleep disorders and inflammatory markers in participants with infertility: Model 1: unadjusted covariates; Model 2: adjusted according to age, marital status, education level, PIR, BMI, and diabetes status.

In addition, subgroup and interaction analyses on age, BMI, diabetes, and hypertension were conducted to ensure the robustness of the results (Y. Zhao et al. 2024), and the statistical significance threshold was set to *p* < 0.05. All analyses were performed via R software (version 4.3.2), the R software packages TwoSampleMR (0.5.5), and MR‐PRESSO (1.0).

## 3 Results

### 3.1 Baseline Characteristics of the Participants

The study included 370 infertile women aged between 20 and 44 years, and the baseline characteristics are compared in Table 1. Age, marital status, education level, PIR, BMI, diabetes status, and PLR were significantly different between the two groups. Among the infertile patients with sleep disorders, the probabilities of being married/living with a partner, having a college education or above, having a middle income level, having a BMI > 30.0 kg/cm^2^, and having diabetes were greater (*p* < 0.05). The average log_2_ PLR of infertile women with sleep disorders was 6.64 × 1000 cells/µL, which was significantly lower than that of infertile women without sleep disorders (*p* = 0.023), but there was no significant difference in the SII, NLR, or LMR levels.

**TABLE 1:** Baseline characteristics of the participants.

Traits	Total	Normal sleep	Sleep disorder	*p*‐value
	*n* = 370	*n* = 279	*n* = 91	
**Age (years)**				**< 0.001** ^*^
20–30	107 (30.2%)	74 (25.0%)	33 (45.2%)	
31–40	181 (49.3%)	142 (54.7%)	39 (34.0%)	
> 40	92 (49.3%)	63 (20.3%)	19 (20.8%)	
**Race**				0.209
Mexican American	62 (12.5%)	44 (12.4%)	18 (12.9%)	
Non‐Hispanic White	124 (56.3%)	91 (54.5%	33 (61.3%)	
Non‐Hispanic Black	90 (13.5%)	62 (12.9%)	28 (15.2%)	
Non‐Hispanic Asian	41 (5.1%)	39 (6.4%)	2 (1.2%)	
Others	53 (12.7%)	43 (13.8%)	10 (9.4%)	
**Marital status**				< 0.001^*^
Married/living with partner	262 (75.4%)	213 (81.6%)	49 (57.6%)	
Divorced/separated/never married/widowed	108 (24.6%)	66 (18.4%)	42 (42.4%)	
**Education**				< 0.001^*^
Below high school	63 (12.5%)	40 (9.2%)	23 (21.7%)	
High School or equivalent	77 (22.3%)	54 (18.0%)	23 (34.7%)	
College or above	230 (65.2%)	185 (72.7%)	45 (43.6%)	
**PIR**				0.001^*^
Low‐income level	124 (25.3%)	83 (21.6%)	41 (35.8%)	
Middle‐income level	130 (36.4%)	95 (32.3%)	35 (48.1%)	
High‐income level	116 (38.3%)	101 (46.1%)	15 (16.1%)	
BMI (kg/cm2)				0.016^*^
< 25.0	99 (25.5%)	78 (27.7%)	16 (19.2%)	
25.0–30.0	67 (18.8%)	54 (21.9%)	13 (9.8%)	
> 30.0	204 (55.7%)	142 (50.4%)	62 (71.0%)	
**Menstrual cycle**				0.099
Regular menstruation	353 (92.1%)	269 (94.2%)	84 (86.3%)	
Irregular menstruation	17 (7.9%)	10 (5.8%)	7 (13.7%)	
**History of pelvic infection**				0.803
Yes	35 (8.1%)	25 (7.8%)	10 (8.7%)	
No	355 (91.9%)	254 (92.2%)	81 (91.3%)	
**Sedentary behavior**				0.471
Yes	193 (51.3%)	147 (52.8%)	46 (52.8%)	
No	177 (48.7%)	132 (47.2%)	45 (47.2%)	
**Physical exercise**				0.080
Yes	364 (98.9%)	276 (99.4%)	88 (2.5%)	
No	6 (1.1%)	3 (6.0%)	3 (97.5%)	
**Smoke**				0.062
Never	244 (62.6%)	193 (64.3%)	51 (57.8%)	
Before	45 (16.0%)	36 (18.0%)	9 (10.2%)	
Now	81 (21.4%)	50 (17.7%)	31 (32.0%)	
**Drink**				0.170
Never	125 (26.7%)	96 (29.0%)	29 (19.8%)	
Before	183 (50.8%)	138 (48.0%)	45 (58.7%)	
Now	62 (22.6%)	45 (23.0%)	17 (21.5%)	
**Diabetes status**				0.021^*^
Yes	38 (10.4%)	20 (7.3%)	18 (80.8%)	
No	332 (89.6%)	259 (92.7%)	73 (19.2%)	
**Hypertension status**				0.642
Yes	59 (14.2%)	42 (13.6%)	17 (15.9%)	
No	311 (85.8%)	237 (86.4%)	74 (84.1%)	
**log_SII**	8.98 (8.48, 9.52)	9.05 (8.50, 9.55)	8.91 (8.47, 9.35)	0.152
**log_PLR**	6.78 (6.46, 7.10)	6.82 (6.61, 7.12)	6.64 (6.23, 7.05)	0.023^*^
**log_NLR**	0.95 (0.54, 1.31)	0.95 (0.55, 1.37)	0.92 (0.43, 1.19)	0.216
**log_LMR**	2.09 (1.85, 2.37)	2.08 (1.81, 2.39)	2.13 (1.85, 2.32)	0.92

*Note*: significance of asterisk and bold value: *p*‐value < 0.05.

### 3.2 Correlation Between Variables and Inflammatory Markers

The single‐factor linear regression analysis in Table 2 revealed a significant correlation between sleep disorders and the PLR. The multivariate regression analysis adjusted for key factors including age, marital status, education level, socioeconomic status, BMI, and diabetes status and revealed that sleep disorders in infertile patients were still significantly correlated with the PLR (*p* < 0.05).

**TABLE 2:** Linear regression analysis between variables and inflammatory markers.

Sleep disorder	Univariate		Multivariate	
	*β* (95% CI)	*p*‐value	*β* (95% CI)	*p*‐value
log_SII	−0.122 (−0.286, 0.042)	0.145	−0.121 (−0.300, 0.057)	0.185
log_PLR	−0.195 (−0.306, −0.085)	< 0.001*	−0.145 (−0.267, −0.023)	0.020*
log_NLR	−0.089 (−0.233, 0.055)	0.226	−0.003 (−0.160, 0.154)	0.967
log_LMR	0.007 (−0.104, 0.119)	0.897	−0.055 (−0.176, 0.067)	0.378

*Note*: The parameters adjusted in multivariable linear regression included age, marital status, education level, PIR, BMI, and diabetes. Significance of asterisk and bold value: *p*‐value < 0.05.

### 3.3 Subgroup Analysis and Interaction Analysis

To further confirm the robustness of the correlation between sleep disorders and the PLR, we also conducted subgroup analysis. As shown in Table 3, the interaction analysis results indicated that hypertension significantly affected the correlation between sleep disorders and log‐PLR (*p* < 0.05). The subgroup analysis results revealed that the levels of PLR maintained a consistently negative association with sleep disorders in BMI > 30 kg/cm^2^, hypertension, or no diabetes subgroups (*p* < 0.05).

**TABLE 3:** The results of subgroup analysis and interaction analysis.

Variables	*β* (95% CI)	*p*‐value	*p*. for interaction
**Age**			0.692
20–30	−0.226 (−0.460, 0.007)	0.075	
31–40	−0.211 (−0.470, 0.049)	0.120	
> 40	−0.061 (−0.314, 0.191)	0.640	
**BMI**			0.857
18.5–25	−0.200 (−0.607, 0.206)	0.346	
25–30	−0.075 (−0.482, 0.333)	0.727	
> 30	−0.204 (−0.355, −0.053)	0.012*	
**Diabetes**			0.817
Yes	−0.236 (−0.584, 0.111)	0.254	
No	−0.193 (−0.353, −0.032)	0.023*	
**Hypertension**			0.040*
Yes	−0.489 (−0.771, −0.208)	0.007*	
No	−0.142 (−0.301, 0.026)	0.104	

*Note*: significance of asterisk and bold value: p‐value < 0.05.

### 3.4 Two‐Sample MR Analysis of 91 Inflammatory Factors and Female Infertility

**FIGURE 3:**
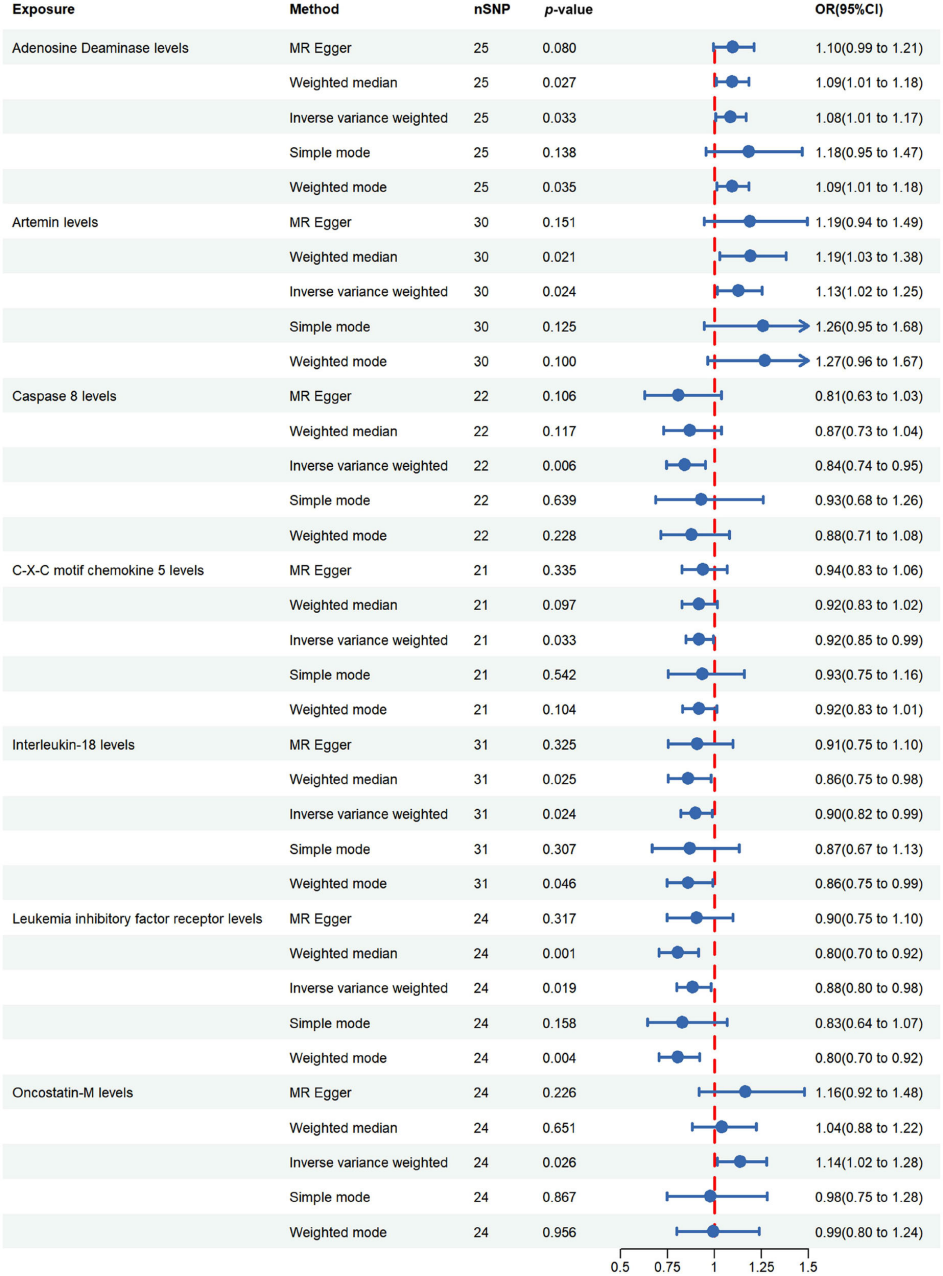
Forest plot of the relationships between seven inflammatory factors and female infertility.

The IVM analysis revealed that seven inflammatory factors were strongly associated with female infertility (Figure 3). The following three inflammatory factors were associated with an increased risk of infertility: adenosine deaminase (ADA) levels (number of SNPs [nSNP] = 25, *p* = 0.033, odds ratio [OR] = 1.084, 95% confidence interval [CI] = 1.006–1.168), artemin levels (nSNP = 30, *p* = 0.024, OR = 1.128, 95% CI = 1.016–1.252), and oncostatin‐M (OSM) levels (nSNP = 24, *p* = 0.026, OR = 1.138, 95% CI = 1.016–1.276). The following four inflammatory factors were significantly associated with a reduced risk of infertility: caspase 8 levels (nSNP = 22, *p* = 0.006, OR = 0.841, CI = 0.743–0.952); C‐X‐C motif chemokine 5 (CXCL5) levels (nSNP = 21, *p* = 0.033, OR = 0.917, 95% CI = 0.846–0.993); interleukin‐18 levels (nSNP = 31, *p* = 0.024, OR = 0.900, 95% CI = 0.821–0.986); and leukemia inhibitory factor receptor (LIFR) levels (nSNP = 24, *p* = 0.019, OR = 0.885, CI = 0.799–0.980) (Supporting Information S3).

The sensitivity analyses revealed the same directional causal estimates: the MR‐PRESSO global test revealed no horizontal pleiotropy in the IVs, the heterogeneity test did not identify abnormal IVs, and the pleiotropy test did not detect horizontal pleiotropy in the IVs (*p* > 0.05). In addition, no single SNP‐driven or biased results were observed in the leave‐one‐out analysis, and the results were not affected by any outliers (Supporting Information S2 and S3).

### 3.5 MR Analyses of Sleep Traits and Seven Infertility‐Related Inflammatory Factors

**FIGURE 4:**
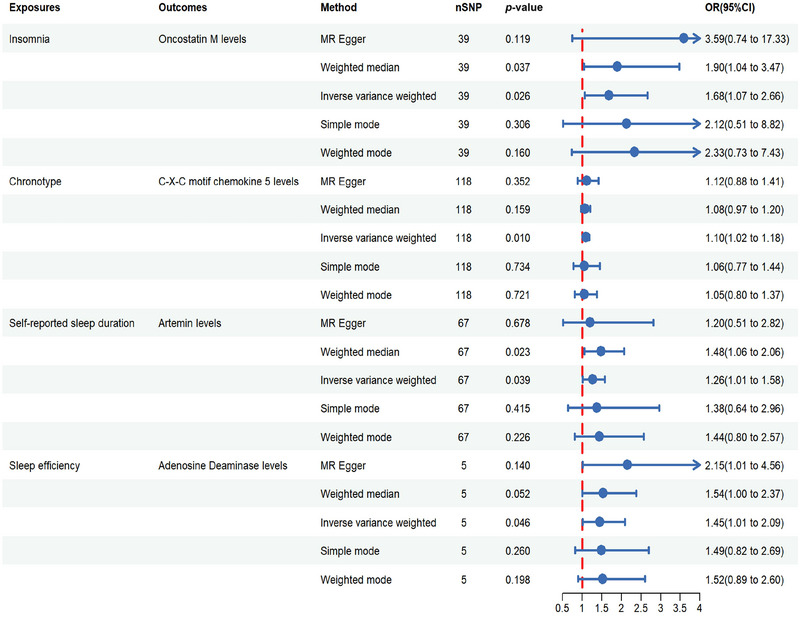
Forest plot of sleep traits and female‐related inflammatory factors.

To further examine whether the 11 sleep traits were causally associated with the seven positive inflammatory factors, we performed two‐sample MR analysis again (Figure 4). Insomnia significantly increased the risk of abnormal OSM levels (*p* = 0.026, OR = 1.683, 95% CI = 1.066–2.660). The morning‐type chronotype significantly increased the risk of the inflammatory factor CXCL5 (*p* = 0.010, OR = 1.100, 95% CI = 1.023–1.182); self‐reported sleep duration was significantly correlated with artemin levels (*p* = 0.039, OR = 1.263, 95% CI = 1.012–1.577); and sleep efficiency was significantly associated with ADA levels (*p* = 0.046, OR = 1.451, 95% CI = 1.007–2.091) (Supporting Information S5). Importantly, no evidence of pleiotropy, heterogeneity, pleiotropy, or sensitivity was observed in the association (*p* > 0.05), and the relevant visualizations are presented in Supporting Information S4 and S5.

### 3.6 Two‐Sample MR Analysis of Sleep Traits and Female Fertility

Neither of the results of the five MR analysis methods revealed a direct causal relationship between sleep traits and female infertility (*p* > 0.05). The results of the horizontal pleiotropy test and heterogeneity test (*p* > 0.05) further enhanced the reliability of the above results. The MR results for sleep traits and infertility are presented in Supporting Information S6.

## 4 Discussion

This study aimed to explore the potential associations among sleep, inflammation, and female infertility via NHANES 2013–2020 survey data and MR analysis. Our results suggested that sleep disorders were significantly associated with lower PLRs in infertile women. The MR analysis results supported the role of inflammation in the genetic association between sleep and infertility.

Analysis revealed that infertile women with sleep disorders tend to have higher education, middle income, high weight, and comorbidities of diabetes. Infertile women exhibiting sleep disorders may have relatively lower levels of PLR, and this association persists even after adjusting for various covariates. The results of the subgroup and interaction analyses further revealed that obesity, diabetes, and hypertension play key roles. A study revealed that the dietary insulin of obese or overweight women significantly affects sleep disorders, which may be related to inflammation and hormones (Mirzababaei et al. 2024), indicating the potential of obesity and glucose metabolism to affect the correlation between sleep and inflammation. Studies have shown that insulin resistance and hypertension are the main causes of infertility among clinical factors, with obesity and a variety of unhealthy lifestyles increasing the risk of delayed pregnancy (Rizvi et al. 2024). Previous studies have shown that inflammatory and immune factors play key roles in a variety of diseases leading to female infertility, and lymphocyte‐related immune imbalance is an important factor affecting pregnancy (Jafarpour et al. 2020). Intrauterine platelet‐rich plasma perfusion therapy has important value for improving thin endometrium and has been applied in the clinic to address infertility (Liu et al. 2024). Previous cross‐sectional studies revealed that the PLR was negatively correlated with infertility, and the increasing trend of the PLR was associated with a lower infertility risk (Y. Chen, Xu, et al. 2024), which may be a representative inflammatory marker of infertility. However, sleep disorders were negatively correlated with PLR levels in infertile women, suggesting that sleep disorders may be involved in the pathogenesis of infertility by affecting PLR levels. The SII, LMR, and NLR are also widely studied inflammatory markers for the development and prognosis of human diseases. SII levels are associated with menopausal symptoms, including sleep difficulties, which may lead to the aggravation of menopausal diseases related to female estrogen deficiency (Korpe et al. 2024). A previous cross‐sectional study of Chinese women with normal pregnancies also revealed the relationship between LMR and women's fertility (Bai et al. 2023). Although no associations between these factors and sleep disorders were found in our study, this may be due to the differences between the populations included.

To obtain more extensive evidence of this association and strengthen the understanding of the association between inflammation and sleep disorders in infertile patients, we included more sleep characteristics and inflammatory proteins for MR analysis. The results of MR analysis showed that inflammatory factors were associated with infertility and that sleep characteristics may increase or reduce the risk of infertility by affecting the levels of related specific inflammatory factors. Although there seems to be no direct genetic causal relationship between sleep characteristics and female infertility, which was consistent with the trend of our first study.

MR results revealed genetic correlations between abnormal ADA, artemin, OSM, caspase 8, CXCL5, interleukin‐18, and LIFR levels and infertility. Related factors have been widely studied in cancer and are believed to be associated with cell proliferation, survival, and invasion (To et al. 2023). However, some studies have also emphasized the association between these inflammatory proteins and reproduction. For example, caspase 8 is often studied in spermatogenesis, while it is also associated with endoplasmic reticulum stress in polycystic ovary syndrome, leading to ovarian dysfunction (Jabarpour et al. 2024); LIFR plays an important role in embryo implantation (Namiki et al. 2023). Our analysis suggested that insomnia increases OSM levels by 68% and that higher OSM levels are associated with an increased risk of infertility. The self‐reported long sleep duration increased the level of artemisinin by 26%, which also led to an increased risk of infertility, whereas sleep efficiency was most closely related to ADA levels, ultimately increasing the risk of infertility by 8.4%. In addition, IVW analysis revealed a significant causal relationship between time type and CXCL5 level. Morning‐type sleep increased CXCL5 expression by 10%, but lower CXCL5 was found to be associated with an increased risk of infertility, indicating that the morning type may help reduce the occurrence of infertility. These results were confirmed by a cross‐sectional study (Liang and Liu, 2022).

Insomnia is caused by psychological pressure and interactions with abnormal autonomous activities (Riemann et al. 2010). Studies have shown that insomnia is related to excessive activation of the hypothalamus–pituitary–adrenal (HPA) axis and the release of large amounts of corticotropin‐releasing hormone and cortisol, thereby affecting reproductive hormones. The severity of insomnia is also positively correlated with the activation of the HPA axis (Kloss et al. 2015). This study revealed that insomnia is related to infertility caused by abnormal reproductive hormone levels; however, the mechanism of the interaction between stress‐related insomnia and infertility needs in‐depth and extensive research. OSM is a member of the serum interleukin‐6 (IL‐6) family, which helps promote the invasion, metastasis, and epithelial mesenchymal transition of tumor cells. Its high expression level is closely related to the occurrence of female reproductive system tumors, such as endometrial cancer and ovarian cancer (Geethadevi et al. 2024). Animal studies have shown that OSM increases the maturation rate of immature oocytes from the MI stage to the MII stage after in vitro fertilization in female mice (Akdemir et al. 2022), suggesting that the role of OSM in female infertility may be related to its role before fertilization. OSM enhances MAPK signal transduction and regulates lipolysis (Dollet et al. 2024). Studies on Drosophila have also reported that MAPK signaling in adipocytes regulates the maturation and excretion of oocytes in early germline cysts (Bradshaw et al. 2024). The latest research has found that OSM may serve as a potential inflammatory biomarker reflecting ovarian function in serum and help reveal changes in follicular fluid during follicular development and progression (C. Wang et al. 2025). These findings provide a possible direction for studying the mechanism by which the insomnia‐induced signaling pathway axis increases the OSM level. Sleep time was significantly correlated with artemin. Previous studies have shown that the overexpression of artemin can predict poor prognosis in patients with endometrial cancer and can be used as an independent risk factor for disease prognosis (X. Wang et al. 2022), and is also believed to be associated with early embryo implantation (Gómez et al. 2017). Insufficient sleep may lead to increased levels of cytokines, such as tumor necrosis factor and IL‐6, as well as immune and inflammatory reactions (Irwin et al. 2006). However, there is no evidence that insufficient sleep can increase the risk of infertility through these inflammatory factors. A cohort study from New York City revealed that sleep time significantly affects women's fertility, which is consistent with our results (Charifson et al. 2023).

Prospective studies have shown that patients who cancel the cycle before oocyte retrieval may have low sleep efficiency during ovulation induction (Pimolsri et al. 2021), which is related to a decline in oocyte quality and the fertilization rate (Huang et al. 2019). We identified a genetic correlation between sleep efficiency and ADA, which provides a new perspective on the mechanism of infertility caused by sleep disorders. Many studies have investigated the genotype–phenotype correlation between ADA levels and male infertility, especially in spermatogenesis (Dai et al. 2023), and it is also associated with insulin resistance in polycystic ovary syndrome, which largely leads to infertility (Öztürk et al. 2019); however, further research is needed to understand how sleep affects the expression of this inflammatory factor and increases the risk of infertility in women.

Previous studies have shown that changes in the endogenous hormone rhythm corresponding to the sleep‐wake cycle are synchronized with changes in the circadian rhythm cycle (Montaruli et al. 2021). A cross‐sectional study revealed that infertile patients had more nocturnal time patterns (Özçelik et al. 2023), which is consistent with our results. Previous studies have focused mostly on analyzing the correlation between symptoms, but this study revealed that the type of nighttime may lead to infertility by affecting the expression of the inflammatory factor CXCL5. CXCL5 is believed to take part in the development of oocytes and embryos in human and mice (Kawagoe et al. 2020). The results of an MR analysis also reflect a genetic association between high levels of CXCL5 and a reduced risk of endometriosis, providing additional evidence for the association between this inflammatory protein and female reproductive diseases (Wei et al. 2024). These findings suggest that late sleep duration may accelerate oocyte and embryo senescence by increasing CXCL5 levels, reducing oocyte quality and embryo development potential, and leading to female infertility; however, the relevant mechanisms need to be further studied.

The results of cross‐sectional and MR studies highlight the potential role of sleep in female infertility through effects on the levels of inflammatory factors. NHANES data were combined with MR analysis data to comprehensively evaluate and adjust various potential confounding factors by using a large sample size. The MR method can help reduce unmeasured confounding factors, reverse causal bias, and improve the reliability of conclusions. These findings are helpful for further understanding the complex influence mechanism and correlation between inflammation, sleep disorders, and infertility; expanding the diagnosis and treatment of female infertility, especially unexplained infertility; and providing guidance for reproductive beneficial sleep regulation behavior in women of childbearing age before and during pregnancy. However, further prospective studies are needed to determine whether there is a more in‐depth, complex, and truly reliable relationship between these factors.

However, this study has several limitations. First, the racial differences involved in the analysis of the American population in the cross‐sectional study and the European population in the MR analysis limit the general applicability of the relevant results to other races or populations. Second, the infertility‐ and sleep‐related characteristics we selected are mainly based on the results of self‐reports, which inevitably leads to classification bias and subjective recall bias, and the sample overlap between GWAS datasets may also lead to bias in causal association estimation. Third, the number of infertility‐related cases is relatively small, and there is a lack of more precise definitions and classifications of the causes of infertility. After repeated detection and correction, the possibility of false positives is not ruled out. Residual and unmeasured confounding factors may also be involved in the relationships among inflammation, sleep, and infertility. In the future, more people should be included to conduct relevant cohort studies and in‐depth biological mechanism experiments to explore more interesting and complex associations between sleep, inflammation, and female infertility.

## 5 Conclusion

In conclusion, our study reveals the associations between sleep, inflammation, and infertility, emphasizing that specific inflammatory factors may provide key information concerning the indirect and complex genetic associations between sleep and reproductive disorders and providing a foundation for future related research. These findings suggest, to some extent, that different sleep characteristics may affect the risk of infertility by regulating the levels of inflammatory factors, emphasizing the need for in‐depth screening and treatment of specific inflammatory factors in infertile patients with sleep problems in the future. Related research will help reveal the mechanisms of infertility and provide evidence for selecting effective treatment methods. It is crucial to pay attention to the inflammation levels of infertile patients with sleep problems and develop personalized lifestyle guidance and treatment plans to achieve pregnancy and maintain reproductive health.

## Author Contributions

**Xin Xin**: writing – original draft, methodology, formal analysis, software, and conceptualization; **Jiaxi Li**: writing – original draft, investigation, visualization, and formal analysis; **Jinfu Zhang**: writing – review and editing, conceptualization, supervision; **Haicui Wu**: writing – review and editing, supervision, project administration, funding acquisition.

## Ethics Statement

The authors have nothing to report.

## Consent

The authors have nothing to report.

## Conflicts of Interest

The authors declare no conflicts of interest.

## Peer Review

The peer review history for this article is available at https://publons.com/publon/10.1002/brb3.70627

## Supporting information




**Supplementary File 1**. Data sources for MR analysis.

**Supplementary File 2**. MR leave‐one‐out sensitivity analysis of 11 sleep traits and 7 infertility‐related inflammatory factors.

**Supplementary File 3**. MR analysis of 91 inflammatory factors and female infertility.

**Supplementary File 4**. MR leave‐one‐out sensitivity analysis of 11 sleep traits and female infertility.

**Supplementary File 5**. MR analysis of 11 sleep traits and 7 infertility‐related inflammatory factors.

**Supplementary File 6**. MR analysis of 11 sleep traits and female infertility

## Data Availability

The data used in this study are publicly available at https://www.cdc.gov/nchs/nhanes. The summary statistics data are publicly available on the IEU OpenGWAS project (https://gwas.mrcieu.ac.uk/). The original contributing data used in the study are fully included in the article and supplementary materials.
