# Supplementary material for: Association of Sleep, Inflammation and Female Infertility: A Cross‐Sectional Survey and Genetic Approach
Source: Brain Behav. 2025 Jun 17;15(6):e70627. doi: 10.1002/brb3.70627 (PMC12171637; doi:10.1002/brb3.70627)
Supplement: Supplementary file 1 — Data sources for MR analysis. [file BRB3-15-e70627-s005.docx]

**Supplementary File 1. Data sources for MR analysis.**

| Phenotype | Sample size | Cases | Controls | Population | Source |
| --- | --- | --- | --- | --- | --- |
| Self-reported sleep duration | 446,118 |  |  | European | UK Biobank |
| Self-reported short sleep | 411,934 | 106,192 | 305,742 | European | UK Biobank |
| Self-reported long sleep | 339,926 | 34,184 | 305,742 | European | UK Biobank |
| Chronotype | 403,195 | 252,287 | 150,908 | European | UK Biobank |
| Daytime napping | 452,633 |  |  | European | UK Biobank |
| Daytime sleepiness | 452,071 |  |  | European | UK Biobank |
| Insomnia | 237,627 | 129,270 | 108,357 | European | UK Biobank |
| Accelerometer-based sleep duration | 84,810 |  |  | European | UK Biobank |
| L5 timing | 85,205 |  |  | European | UK Biobank |
| Sleep efficiency | 84,810 |  |  | European | UK Biobank |
| The number of sleep episodes | 84,441 |  |  | European | UK Biobank |
| 91 inflammation factors | 14,824 |  |  | European | UK Biobank |
| Infertility | 75,450 | 6,481 | 68,969 | European | FinnGen |
