# Supplementary figures and images for: Association of Sleep, Inflammation and Female Infertility: A Cross‐Sectional Survey and Genetic Approach
Source: Brain Behav. 2025 Jun 17;15(6):e70627. doi: 10.1002/brb3.70627 (PMC12171637; doi:10.1002/brb3.70627)

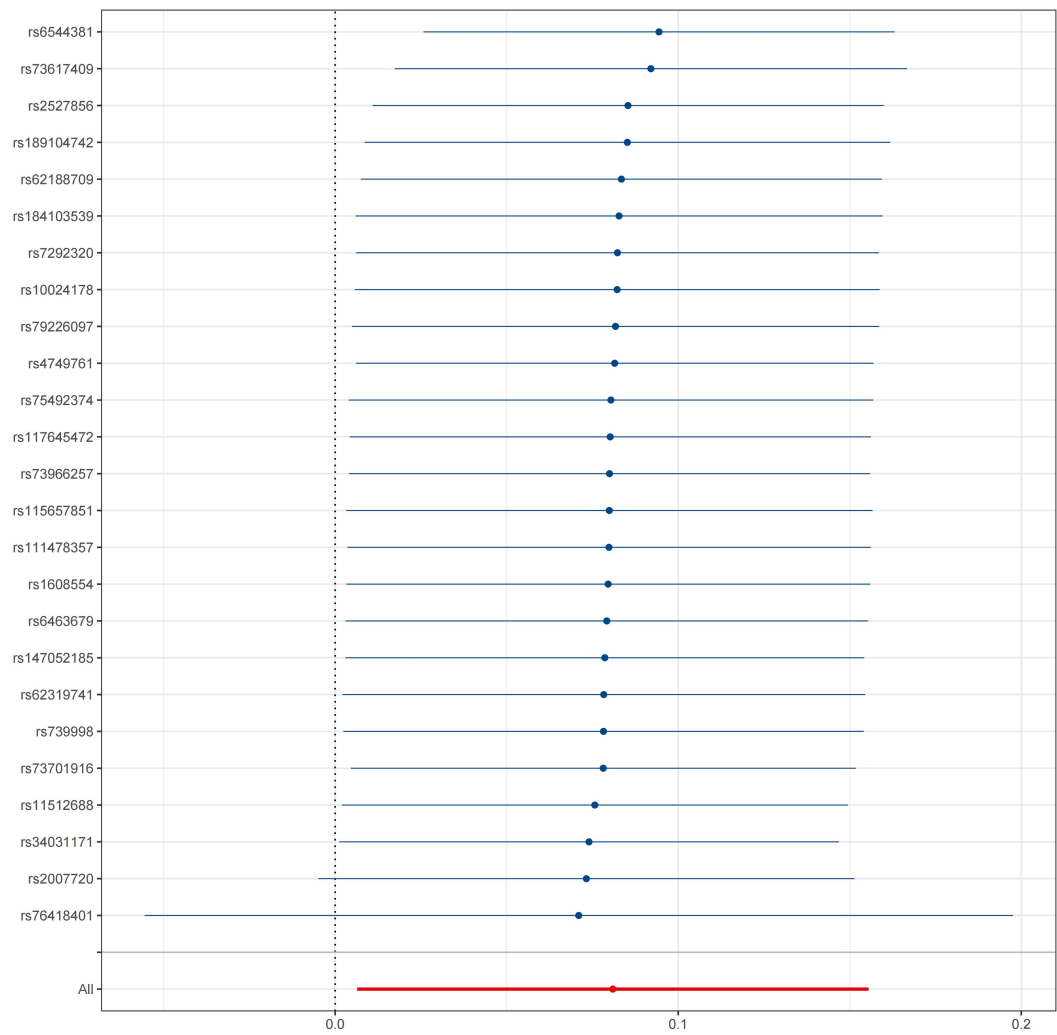

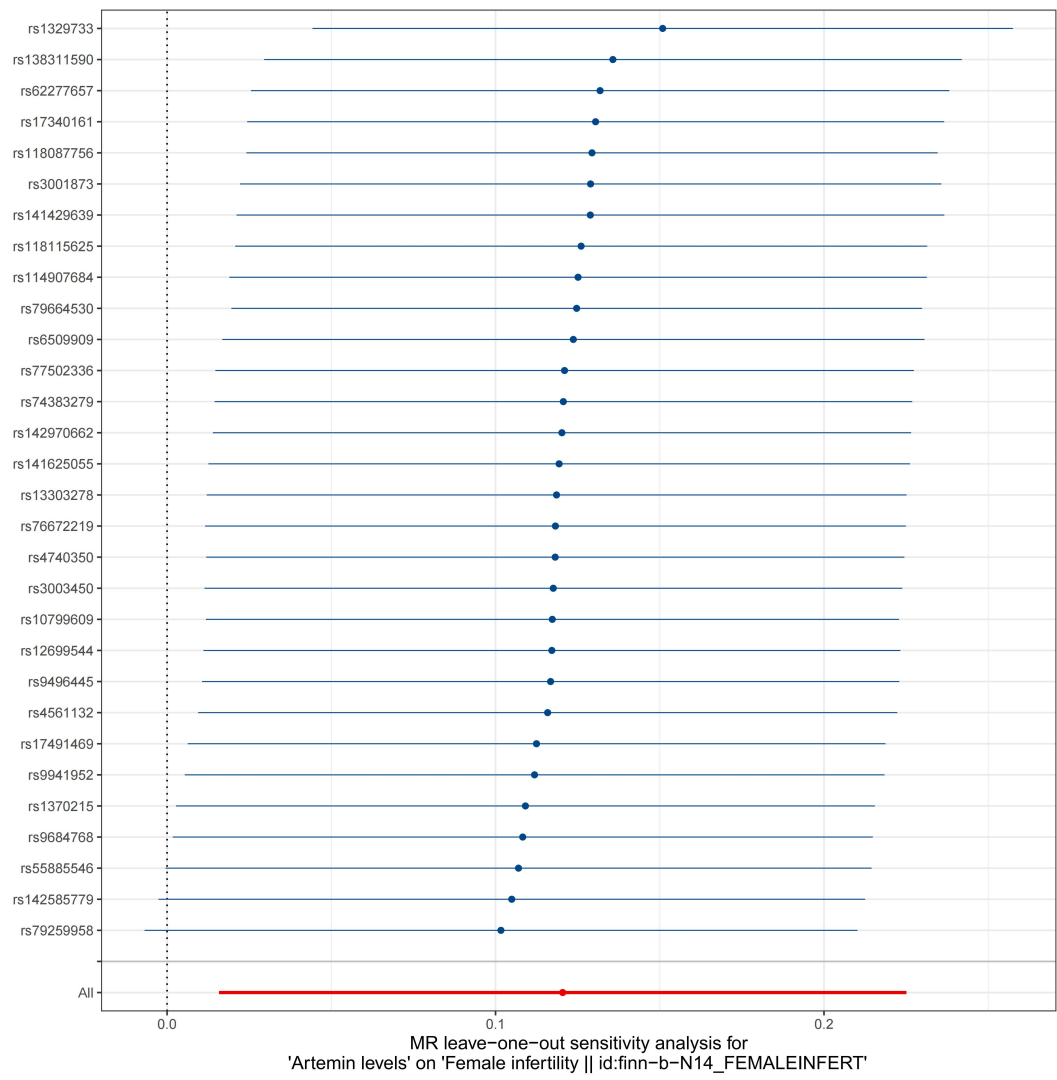

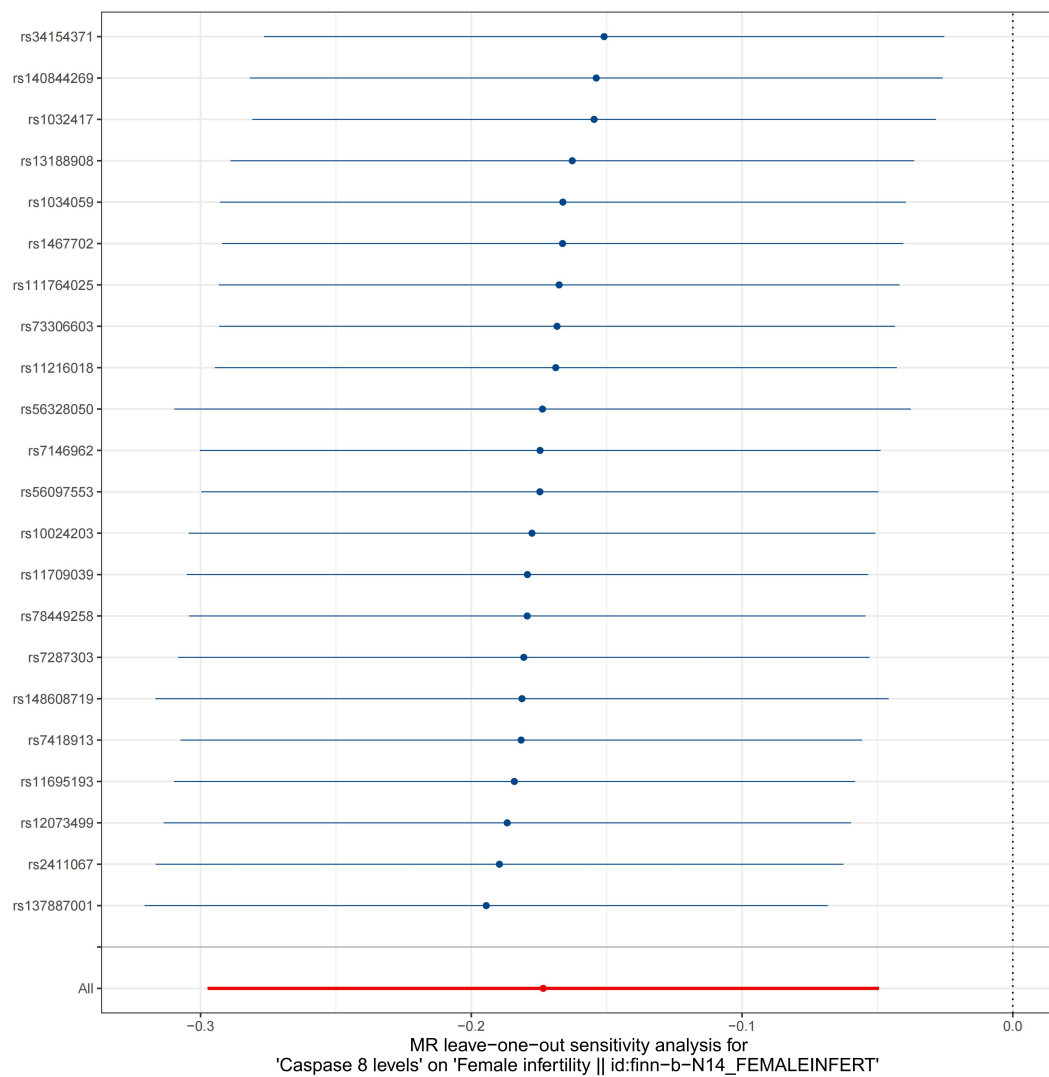

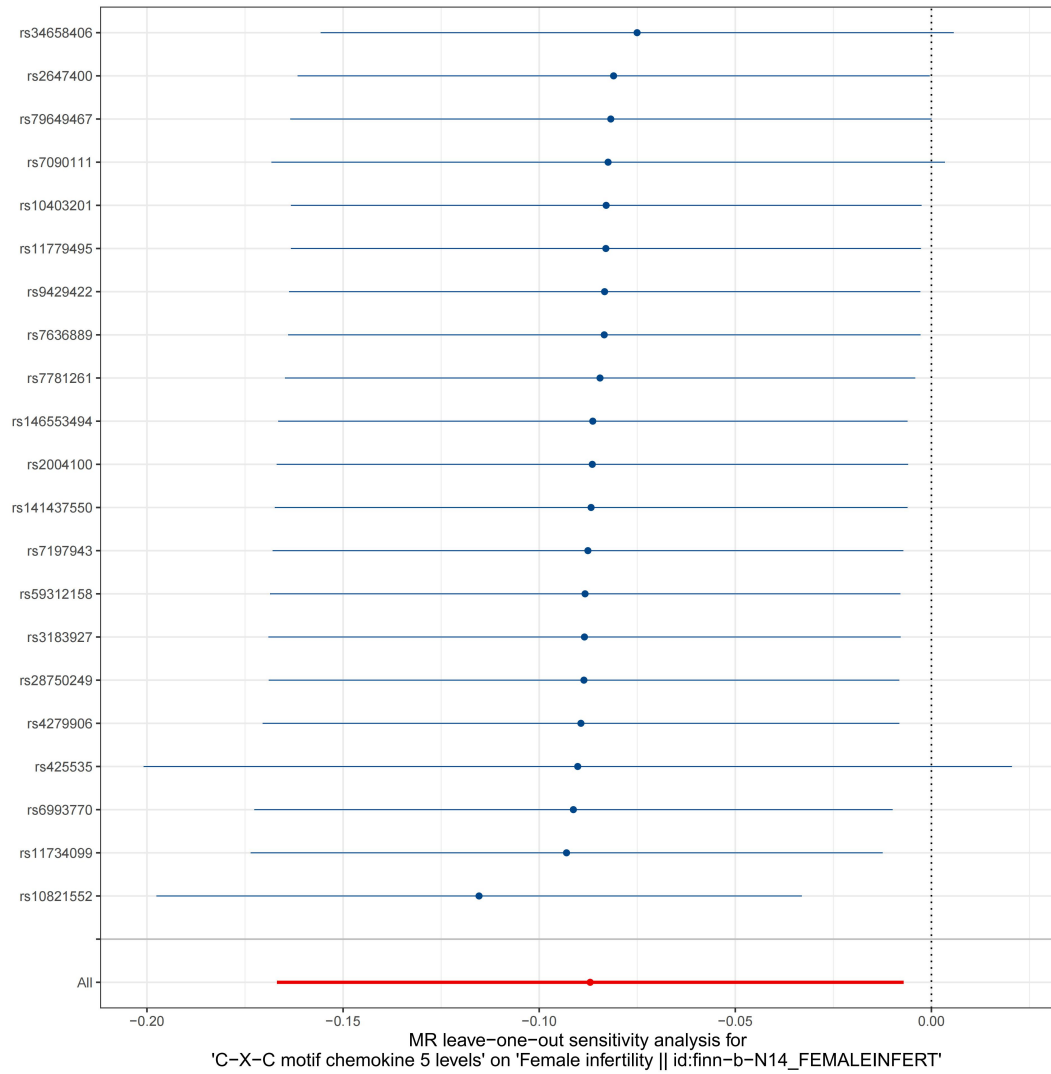

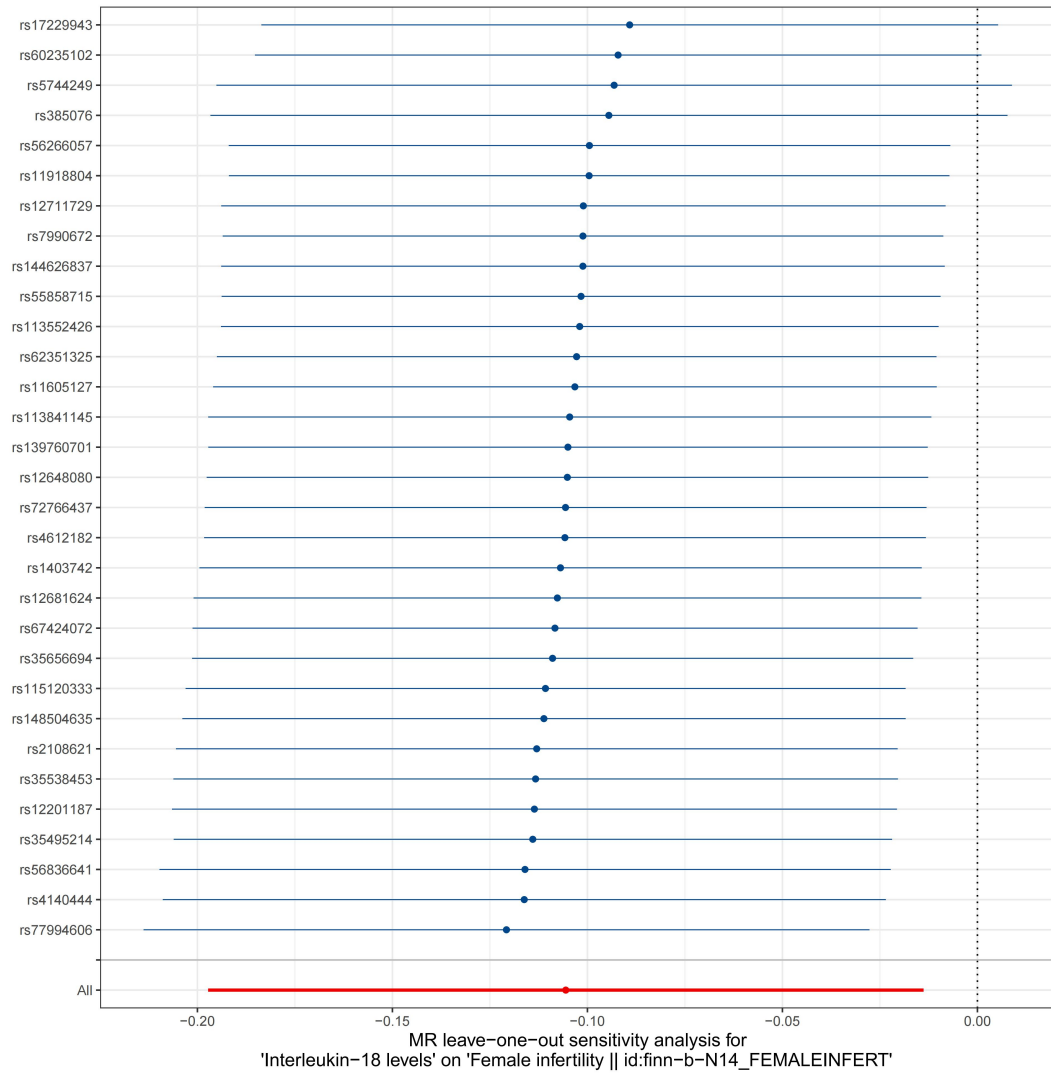

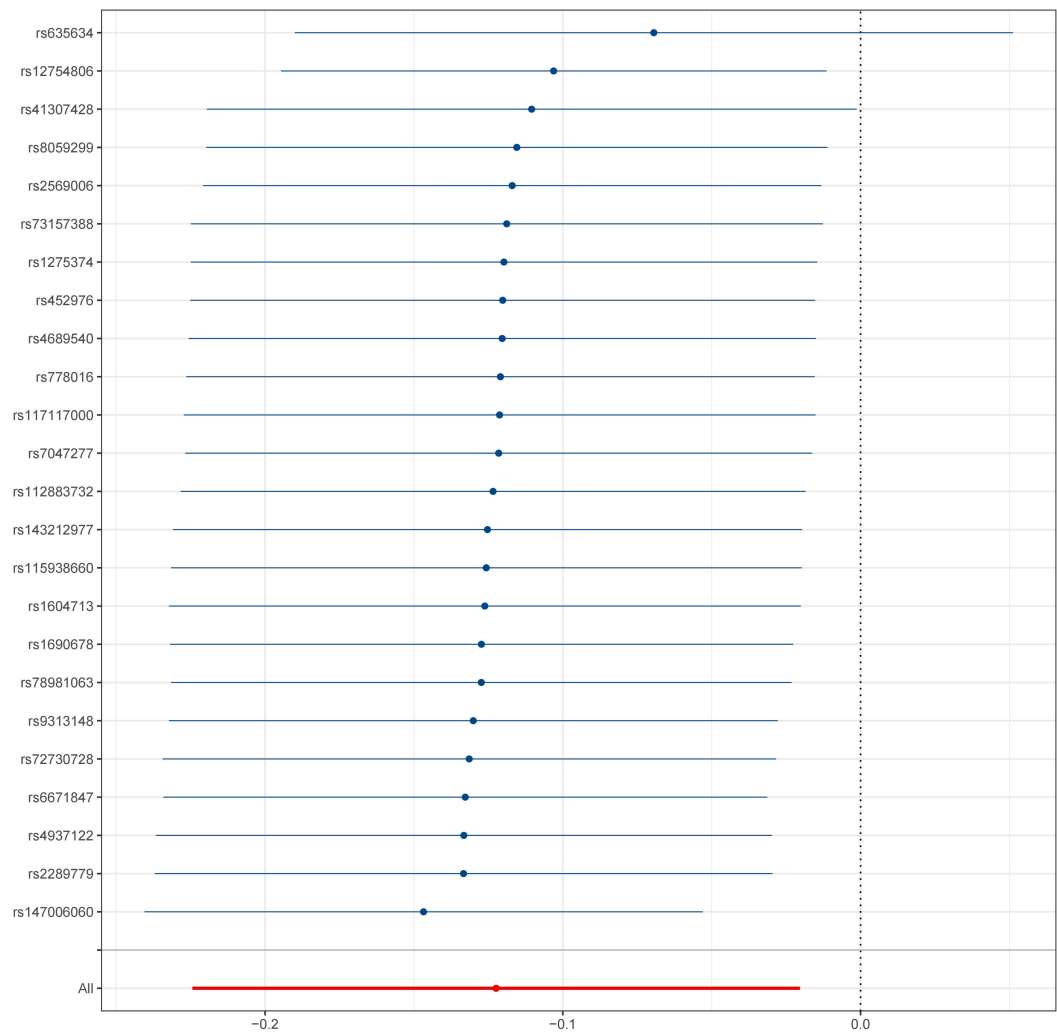

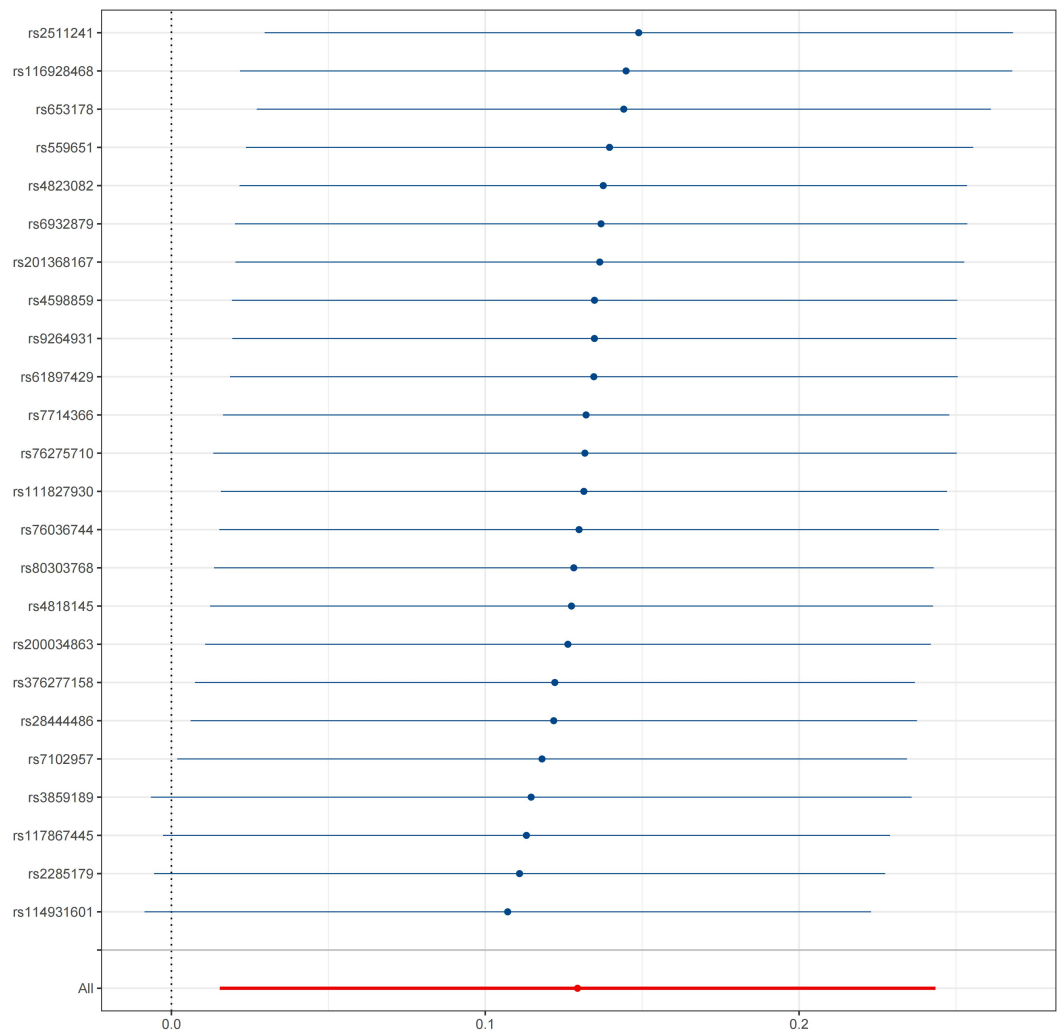

Supplement: Supplementary file 2 — MR leave‐one‐out sensitivity analysis of 11 sleep traits and 7 infertility‐related inflammatory factors. [file BRB3-15-e70627-s001.pdf]

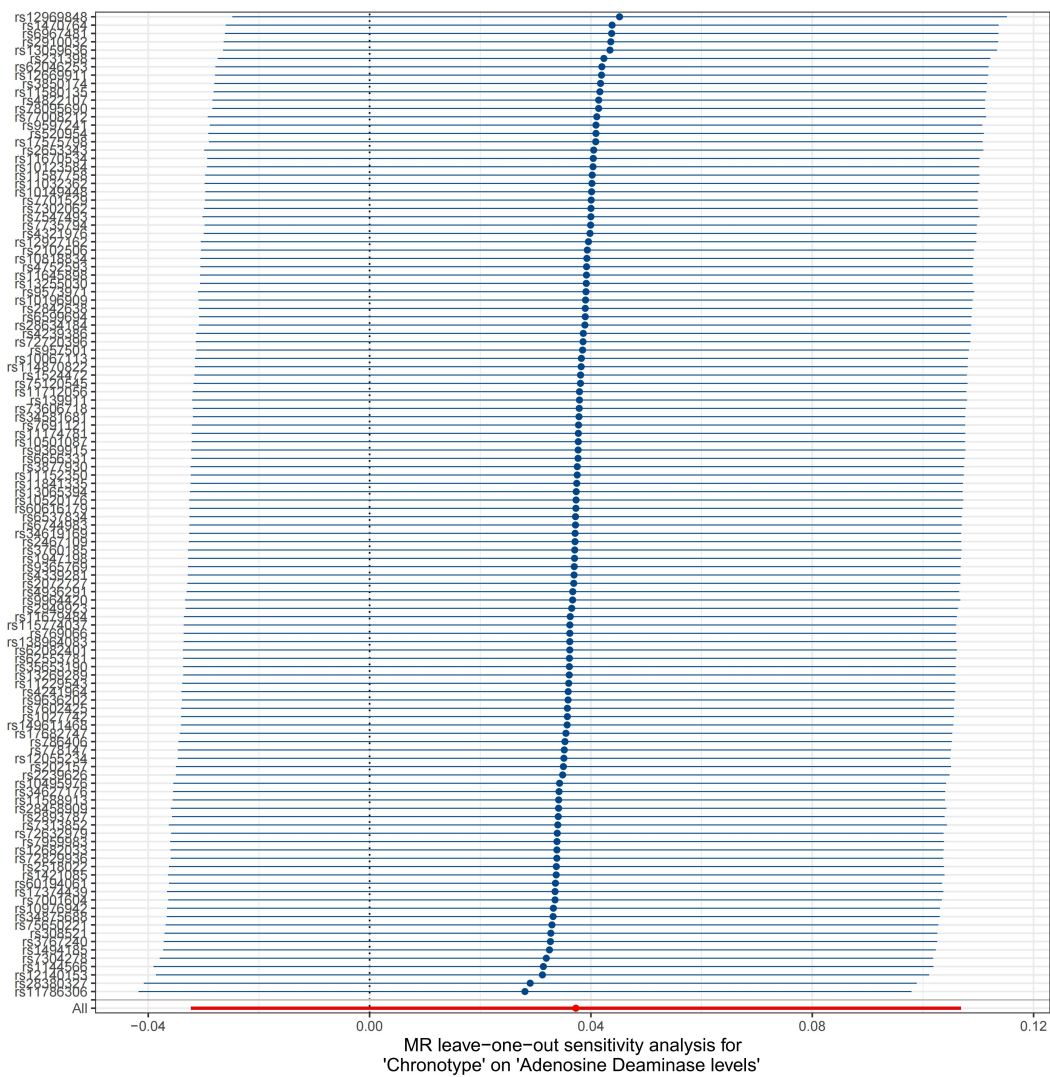

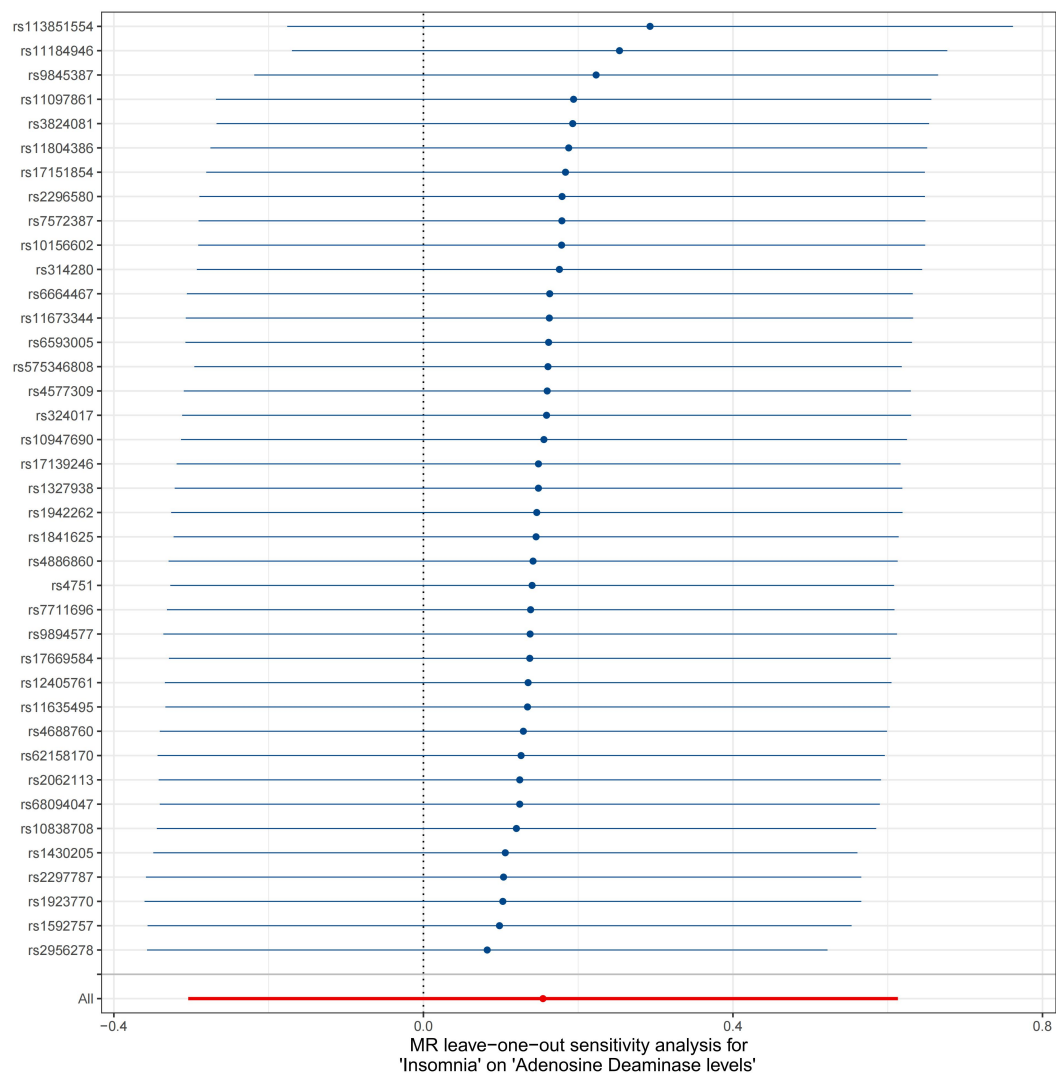

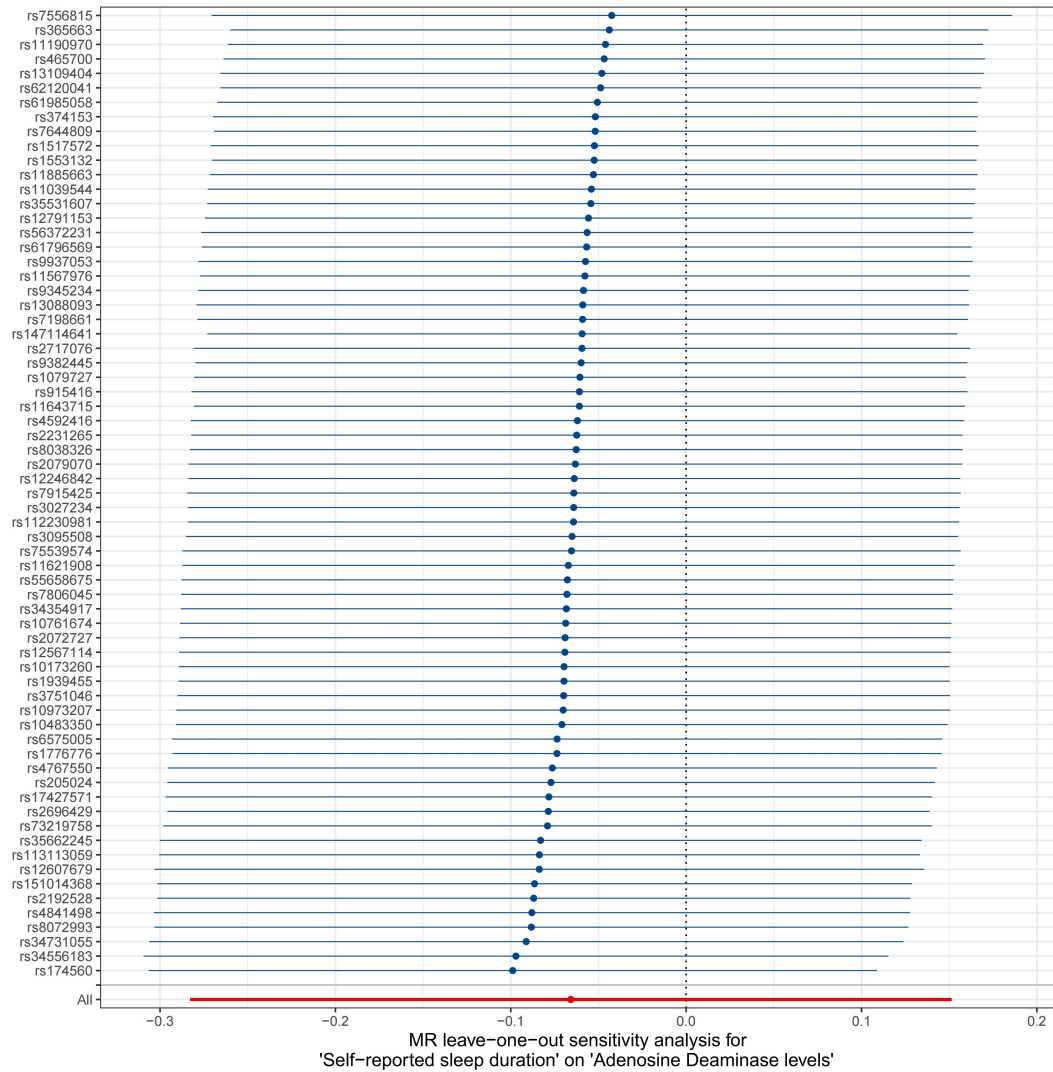

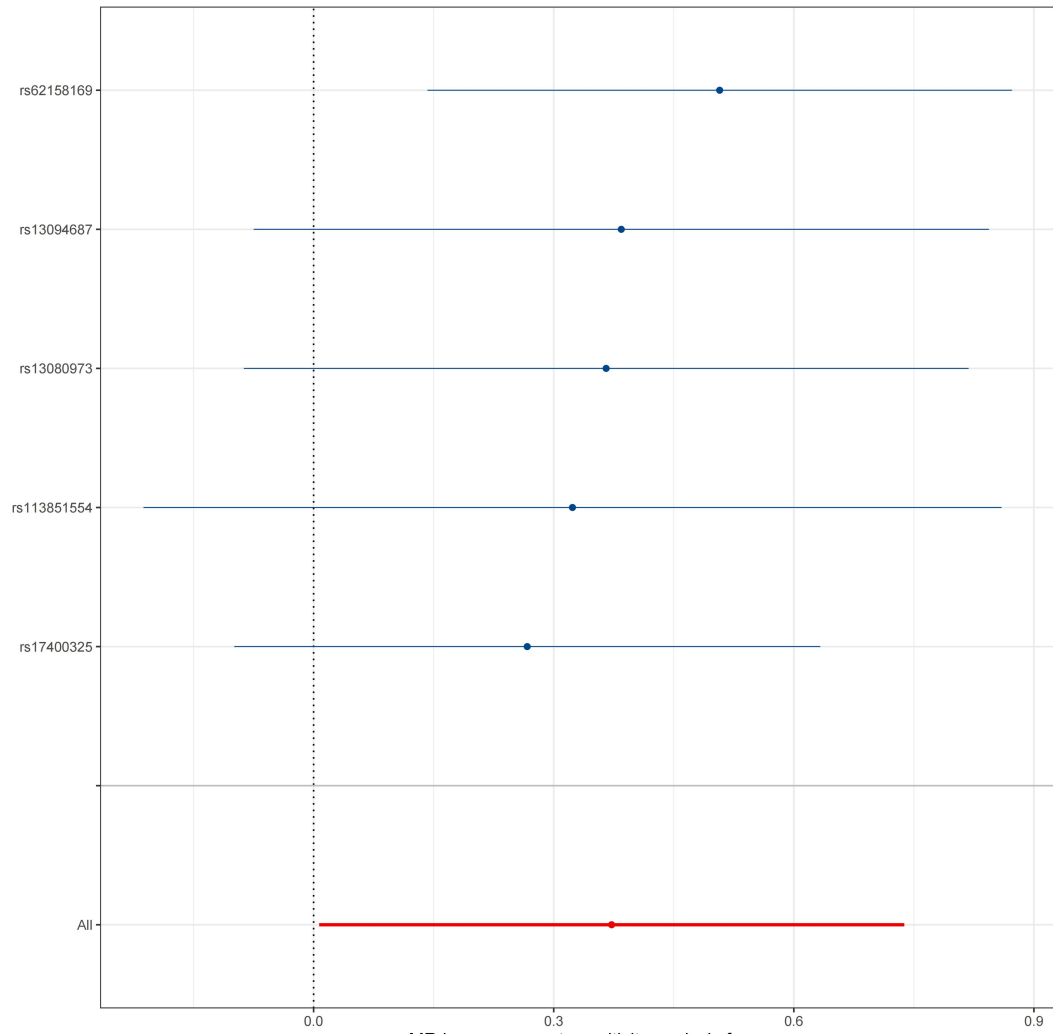

MR leave-one-out sensitivity analysis for  
'Sleep efficiency' on 'Adenosine Deaminase levels'

Supplement: Supplementary file 4 — MR leave‐one‐out sensitivity analysis of 11 sleep traits and female infertility. [file BRB3-15-e70627-s003.pdf]
